# Preliminary Comparison of Oral and Intestinal Human Microbiota in Patients with Colorectal Cancer: A Pilot Study

**DOI:** 10.3389/fmicb.2017.02699

**Published:** 2018-01-12

**Authors:** Edda Russo, Giovanni Bacci, Carolina Chiellini, Camilla Fagorzi, Elena Niccolai, Antonio Taddei, Federica Ricci, Maria N. Ringressi, Rossella Borrelli, Filippo Melli, Manouela Miloeva, Paolo Bechi, Alessio Mengoni, Renato Fani, Amedeo Amedei

**Affiliations:** ^1^Immunology, Department of Clinical and Experimental Medicine, University of Florence, Florence, Italy; ^2^Department of Biology, University of Florence, Florence, Italy; ^3^Department of Surgery and Translational Medicine, University of Florence, Florence, Italy; ^4^Neuromusculoskeletal Department (Interdisciplinary Internal Medicine), Azienda Ospedaliera Universitaria Careggi, Florence, Italy

**Keywords:** colorectal cancer, gut microbiota, oral microbiota, *Fusobacterium nucleatum*, quantitative polymerase chain reaction, taxonomic analysis

## Abstract

In this study Next-Generation Sequencing (NGS) was used to analyze and compare human microbiota from three different compartments, i.e., saliva, feces, and cancer tissue (CT), of a selected cohort of 10 Italian patients with colorectal cancer (CRC) vs. 10 healthy controls (saliva and feces). Furthermore, the *Fusobacterium nucleatum* abundance in the same body site was investigated through real-time quantitative polymerase chain reaction (qPCR) to assess the association with CRC. Differences in bacterial composition, *F. nucleatum* abundance in healthy controls vs. CRC patients, and the association of *F. nucleatum* with clinical parameters were observed. Taxonomic analysis based on 16S rRNA gene, revealed the presence of three main bacterial phyla, which includes about 80% of reads: *Firmicutes* (39.18%), *Bacteroidetes* (30.36%), and *Proteobacteria* (10.65%). The results highlighted the presence of different bacterial compositions; in particular, the fecal samples of CRC patients seemed to be enriched with *Bacteroidetes*, whereas in the fecal samples of healthy controls *Firmicutes* were one of the major phyla detected though these differences were not statistically significant. The CT samples showed the highest alpha diversity values. These results emphasize a different taxonomic composition of feces from CRC compared to healthy controls. Despite the low number of samples included in the study, these results suggest the importance of microbiota in the CRC progression and could pave the way to the development of therapeutic interventions and novel microbial-related diagnostic tools in CRC patients.

## Introduction

Colorectal cancer (CRC) is the third most frequent tumor worldwide and is considered as the fourth leading cause of cancer deaths (Rowland, 2009), accounting for about 1.2 million new cases and 600,000 deaths per year. The CRC etiology is still not fully understood, but the development of colorectal carcinogenesis is a heterogeneous progression with different sets of genetic and epigenetic variations, and is influenced by dietary patterns, environmental conditions, host immunity, and microbial adhesion (as reviewed in Russo et al., 2016). A large number of microbes is able to live and thrive in the human gut, forming huge and complex communities, which, in turn, may play key roles in the CRC development (Warren et al., 2013; Gao et al., 2017).

Also, the association between some bacteria with the initiation and progression of neoplasia, e.g., *Helicobacter pylori* to gastric cancer (Amedei et al., 2014), has been well-established. This infection is the cause of approximately 5.5% of all cancers (Parkin, 2006). Whilst the fine processes of cancerogenesis have not been fully elucidated, it is reasonable to prevent its risk by targeting the possible microbial etiologic agent(s), as previous studies have suggested a potential dysbiosis of gut microbiota in CRC patients (Akin and Tözün, 2014). Many works have detected the hypothetical pathogens linked to CRC pathogenesis, aimed to improve CRC prevention and treatment (as reviewed in Russo et al., 2016). In the last years, the 16S rRNA gene sequencing approach has been widely used as an effective tool to analyze the complex microbial community (Eckburg et al., 2005). The obtained results suggested that the breakdown of the intestinal microbiota structure could promote carcinogenesis and CRC development.

Other studies of CRCs reported the presence of links between the intestinal microbiota and bacterial metabolites and the tumor development in murine colitis-associated CRC models (Gao et al., 2015; O'Keefe, 2016). Indeed, fermenting bacteria may produce protective molecules in the colon such as butyrate, the conjugate base of butyric acid, which has been shown to exert a beneficial effect on colon cells (Lupton, 2004; Pessione, 2012). On the other hand, the antibiotic suppression of intestinal microbiota has been shown to reduce the lipoperoxidation associated with the proliferation of colon carcinogenesis in murine models (Martin et al., 2015).

The presence of specific microorganisms in the microbiota and the subsequent inflammation (often microbial driven) and cancer are linked (Mantovani, 2009; Ben-Neriah and Karin, 2011). The inflammatory tumor process is actually added as a distinct cancer hallmark (Hanahan and Weinberg, 2011). Recent studies suggested that both the whole colon microbiota and specific pathogenic microbial strains likely play a crucial role in CRC development, in both direct and indirect ways (Candela et al., 2014; Lin and Zhang, 2017). In this context, *Fusobacterium nucleatum* appears to be particularly interesting. Indeed this nonspore-forming, anaerobic gram-negative oral commensal bacterium, is linked to periodontal disease, but several reports have shown a correlation with a wide range of disorders, including gastrointestinal and cardiovascular diseases, rheumatoid arthritis, respiratory tract infections, and Alzheimer's disease (for a review see Han, 2015). Furthermore, it is increasingly emerging that members of *Fusobacterium* genus, in particular *F. nucleatum*, inhabit stool and tumor tissues samples of patients affected by the CRC and colorectal adenomas (Ito et al., 2015). On the other hand, few studies suggested a correlation between *F. nucleatum* and colon cancer invasiveness (e.g., lymph node metastasis); however, this hypothesis has not been completely corroborated (Castellarin et al., 2012; Flanagan et al., 2014), even though an enrichment of *F. nucleatum* in CRC tissue has been detected, by both metagenomic and qPCR analyses of DNA from feces (Kostic et al., 2012).

In this study we used targeted metagenomics based on 16S rRNA gene to deeply explore three different human districts trying to define possible microbial markers of CRC not exclusively related to the tumor lesion.

## Materials and methods

### Patients

In total, 10 Italian patients (four males and six females with an age range of 71–95 years) with colorectal adenocarcinoma (confirmed by histological analysis), undergoing surgical resections at the Unit of Surgery, University Hospital of Careggi (AOUC), University of Florence, between October 2015 and March 2016 and 10 healthy controls (six males and four females with an age range of 63–86 years) were recruited. The patients with other CRCs, or exposed to antibiotic therapy within 3 months prior to sample collection, as well those who have undertaken radiotherapy and chemotherapy before the surgical resection and those with comorbid malignancies of other organs were excluded. The 10 healthy subjects were selected based on age, and body mass index, no gastrointestinal disorders, no antibiotic use during the 3-month period prior to sample collection. All the 10 CRC patients were divided according to the pathological TNM (tumor-Node-Metastasis) staging system 7th edition and Dukes classification (Williams and Beart, 1992). The TNM classification uses three parameters to divide the patients into different stages: depth of tumor penetration into the gastric wall (T parameter), the number of metastatic regional lymph nodes involved (N parameter) and the presence of distant metastases (M parameter). Data collected included anthropometric measurements (height, weight), nutritional data (including the use of probiotics), clinical history and status and medication history (Table 1 and Table S1).

**Table 1:** Clinical parameters of CRC patients (CM) and healthy controls (CFP).

**Code**	**Age range**	**Diagnosis**	**TNM**	**Tumor site**	**Height (m)**	**Weight (Kg)**	**BMI**
CM7	75–80	1	pT3 N0 Mx	Colon	1.55	50	20.81
CM8	75–80	2	pT1 N0	Colon	1.57	60	23.34
CM10	75–80	1	pT3 N2a Mx	Colon	1.70	70	24.22
CM11	85–90	1	pT3 N0 Mx	Colon	1.70	67	23.18
CM18	80–85	1	pT3 N1b Mx	Colon	1.80	87	26.85
CM19	95–100	3	pT3 N1b Mx	Colon	1.60	62	24.22
CM20	75–80	1	pT1 N0	Colon	1.75	55	17.96
CM22	80–85	1	pT3 N1a Mx	Colon	1.63	53	19.95
CM23	75–80	2	pT2 N0	Rectum	1.66	67	24.31
CM24	70–75	2	pT3 N2b M1	Sigma-Rectum	1.59	86	34.02
CFP1	80–85				1.66	59	21.41
CFP2	70–75				1.80	82	25.31
CFP3	80–85				1.73	100	33.41
CFP4	75–80				1.70	64	22.15
CFP6	80–85				1.68	62	21.97
CFP7	60–65				1.68	65	23.08
CFP8	70–75				1.86	94	27.17
CFP9	80–85				1.80	82	25.31
CFP10	70–75				1.88	76	21.50
CFP11	80–85				1.70	85	29.41

*TNM (Tumor-Node-Metastasis) staging system 7th (Williams and Beart, 1992). BMI, Body Mass Index; Diagnosis: 1 = Intestinal Adenocarcinoma medium differentiate; 2 = Intestinal Adenocarcinoma moderate differentiate; 3 = Tubulo-villous adenoma with intestinal epithelial dysplasia; Put underneath ≪ Patients ≫ in Materials and methods*.

Unstimulated saliva and stool were collected from both patients and healthy controls, while CT samples were collected only from CRC patients. The day before surgery, in the early morning, saliva was collected by spitting after 1 min without swallowing into a sterile tube; stool samples were collected in a sterile container. CT samples were collected in sterile conditions during surgery. The oral health status of the patients was assessed as it can have a major impact on the salivary microbiota (Table S1). For ethical reasons, we collected only saliva and stool samples from healthy controls. After collection, saliva and stool samples were immediately frozen and stored at −80°C until DNA extraction. Fresh CRC tissues from each patient were collected in physiological solution (NaCl 0.9%). Samples were stored at −80° until use. The study protocols were approved by the ethics committee of AOUC Careggi and complied with the “Declaration of Helsinki.” A written approval was obtained from each participant.

### Bacterial DNA extraction

DNA was extracted from all samples using PowerLyzer® PowerSoil® DNA Isolation Kit (MO BIO laboratories, Inc., Carlsbad, California, USA) with some modifications to the manufacturer's instructions for fecal and saliva samples (Gao et al., 2015).

The modifications were performed on fecal samples as follows (Wesolowska-Andersen et al., 2014): 0.5 g of each stool sample was treated with 2.5 ml of PowerSoil® Bead Solution. Samples were vortexed and then centrifuged at room temperature for 5 min at 1,500 × g. 1 ml of the supernatant was transferred to a PowerLyzer® Glass Bead Tube and 750 ml of PowerSoil® Bead Solution were added. Each sample was incubated for 10 min at 65°C and then for 10 min at 95°C. By this step, manufacturer's instructions were followed.

The modifications on saliva samples were performed as follows (Aagaard et al., 2013): 2 ml of each saliva sample was centrifuged at room temperature for 15 min at 2,600 g. The pellet was resuspended with 750 μl of the PowerSoil® Bead Solution. Then, the sample was loaded into the PowerLyzer® Glass Bead Tube according to the manufacturer's instructions.

Quantity and purity of extracted DNA were checked by 0.8% agarose gel electrophoresis in Tris-EDTA buffer. Each sample was then quantified with the Qubit fluorometer (Life Technologies). All DNA samples were stored at −20°C until used.

### 16S rRNA gene analysis

Extracted DNA samples were sent to an external company (IGA Technology Services-Udine-Italy) for library construction and sequencing on the MiSeq Illumina platform with paired-end protocol. In particular, the V3-V4 region of bacterial 16S rRNA gene was amplified *via* PCR using specific primers (806R and 515F) as previously described (Checcucci et al., 2016). Raw sequences were processed following the UPARSE pipeline (Edgar, 2013). First, sequences were quality refined using StreamingTrim 1.0 (Bacci et al., 2014). Low quality segments were removed using a quality cutoff of 18 Phreds whereas constructs that might have been generated during sequencing procedures, were removed only if found in the top 50 residues of each sequence. Mate pairs were subsequently assembled using the “fastq_mergepairs” command of the USEARCH suite (Edgar et al., 2011). About 92% of the initial pairs were correctly merged, collecting more than 12 million sequences. Sequences were additionally filtered using the “fastq_filter” command of the USEARCH suite with a maximum error rate of 1.0 and a fixed length of 300 bp. Identical sequences were then merged (de-replication) and singleton sequences, namely those found only one time, were removed to reduce errors due to the amplification process (Morgan et al., 2014). De-replicated sequences with an identity higher than 97% were pooled together using the “cluster_otus” commands of the USEARCH suite. Putative chimeric sequences were automatically detected during this step, but an additional chimeric identification step was also included, using the “uchime_ref” command (Edgar et al., 2011) in combination with the latest RDP training set available (trainset16 02/2016) (Cole et al., 2014). Finally, 17′971 representative sequences were collected and used as reference for the clustering step that was performed using the “-usearch_global” command of the USEARCH suite with a sequence identity threshold of 97% (corresponding to the species level according to Konstantinidis and Tiedje (2007). More than 72% of the initial sequences were correctly assigned to an OTU (8′595′385 sequences). For each OTU (cluster), a single representative sequence was used for taxonomic annotation. Sequences were classified using the SINA standalone classifier in combination with the “Ref NR 99” database (release 123) (Pruesse et al., 2012). Clusters classified as “Eukaryota” were removed along with unclassified clusters retaining a set of 2,386 clusters classified as “Bacteria” or “Archaea.” A detailed description of the number of sequences collected at each step of analysis is reported in Table S2.

### Real-time quantitative PCR

To estimate the relative amount of *F. nucleatum* over the total amount of bacteria, the DNA from each sample was assayed by real-time quantitative PCR (qPCR); the estimation of the total number of 16S rRNA gene copies in all samples was performed with bacterial primers Eub341F (5′- CCTACGGGAGGCAGCAG-3′) and Eub515R (5′-TACCGCGGCKGCTGGCA-3′) targeting the 16S rRNA gene, using a previously reported protocol (Abdelrhman et al., 2016); the value of *F. nucleatum* was assessed with specific primers: fuso-F (5′-CTTAGGAATGAGACAGAGATG-3′) and fuso-R (5′-TGATGGTAACATACGAAAGG-3′) (Periasamy and Kolenbrander, 2009), targeting a fragment of 16S rRNA gene. qPCR was performed in an QuantStudio™ 7 apparatus (Applied Biosystems), following the amplification program described in Periasamy and Kolenbrander (2009). *F. nucleatum* DSM 20482 (ATCC 10953) DNA was used as standard for qPCR quantification. Reactions were performed in triplicates in 10 μl final volume as described in Checcucci et al. (2016). qPCR results were analyzed by comparing the Cq values of the samples, representing the threshold cycles; Cq is a relative measure of the concentration of the target gene in the PCR reaction; lower Cq values indicate high amounts of targeted nucleic acid, while higher Cq values indicate lower amounts of the target nucleic acid. The presence of *F. nucleatum* has been calculated as the ratio between the Cq value of *F. nucleatum* 16S rRNA gene and the Cq value of the total bacterial community 16S rRNA gene amplicons. The results were analyzed with the Mann-Whitney pairwise *post-hoc* tests, using the PAST3 Software (Hammer et al., 2001).

### Statistical analysis

Statistical analyses on the bacterial community distribution were implemented in R (R Core Team, 2014) using the vegan package (version 2.3-2) (Oksanen et al., 2015). Analysis of similarity (ANOSIM, “anosim” function) was conducted to test the statistical significance of difference between distinctive bacterial communities.

Microbiome dataset was analyzed in R (R Core Team, 2014) with the help of additional libraries as reported below. Accumulation curves were calculated using the “specaccum” function (vegan package version 2.4) (Oksanen et al., 2015) adding sites in random order with 100 permutations for each step. Differences in bacterial population structure between different body sites were inspected using the “anosim” function (vegan package) with 1,000 permutations and plotted using the ggplot2 library (version 2.2) (Wickham, 2009). Hierarchical clustering of bacterial distribution was performed based on the average linkage method with Bray-Curtis dissimilarity indexes (“vegdist” function, vegan package) and visualized using custom scripts. OTU counts were normalized for each sample by dividing them by the total counts of all OTUs within that sample. Differences in the abundance of OTUs assigned to *Fusobacterium* genus between CRC and healthy patients were inferred using Student's *t*-test on normalized data (“*t*-test” function of the R stats package). The distribution of *Fusobacterium* representatives in biopsy samples of CRC patients was modeled using linear models and one-way analysis of variance (“lm” and “aov” functions of stats Package) on quantitative and qualitative factors, respectively. The LEfSe pipeline (Segata et al., 2011) was performed on OTU count collapsed according to shared taxonomic classifications to identify distinctive taxa of each body site here explored (saliva, feces, and biopsy). Differentially abundant OTUs were detected using differential abundance analysis with a zero-inflated log-normal model as implemented in the “fitFeatureModel” function of the metagenomeSeq R package, version 1.16 (Paulson et al., 2013).

Shannon, Chao 1, Evenness and Richness indices were used to estimate bacterial diversity in each sample (Tables S6, S7). The indices were computed using the “diversity” function of the vegan R package version 2.4. The percentage of coverage was calculated by Good's estimator (Good, 1953) using the formula: [1—(n/N)] × 100, where n is the number of sequences found once in a sample (singletons), and N is the total number of sequences in that sample. The Evenness index was calculated using the formula E = S/log(R), where S is the Shannon diversity index and R is the number of OTUs in the sample (the Richness). Different alpha diversity values were tested using mixed-effect model since our data contained observations that were not necessarily independent of one another, namely the patients (West et al., 2014). The “lme” function of the nlme R package (version 3.1) (Pinheiro et al., 2016) was used to construct a model for each diversity index measured (Table S3) to inspect different diversity level across different environment and between healthy and CRC patients. A random intercept model was built using subject ids as a random effect for each index considered.

### Accession number

The 16S rRNA gene sequence data generated in this study was submitted to the GenBank Sequence Read Archive accession number PRJNA356414.

### Ethics approval and consent to participate

The study was reviewed and approved by AOUC Careggi Institutional Review Board (Prot 2010/0012462). All study participants, or their legal guardian, have provided an informed written consent prior to the study enrollment in compliance with national legislation and the Code of Ethical Principles for Medical Research Involving Human Subjects of the World Medical Association (Declaration of Helsinki).

## Results

### Overall comparison of the oral, fecal, and cancer tissue microbiota

**Figure 1:**
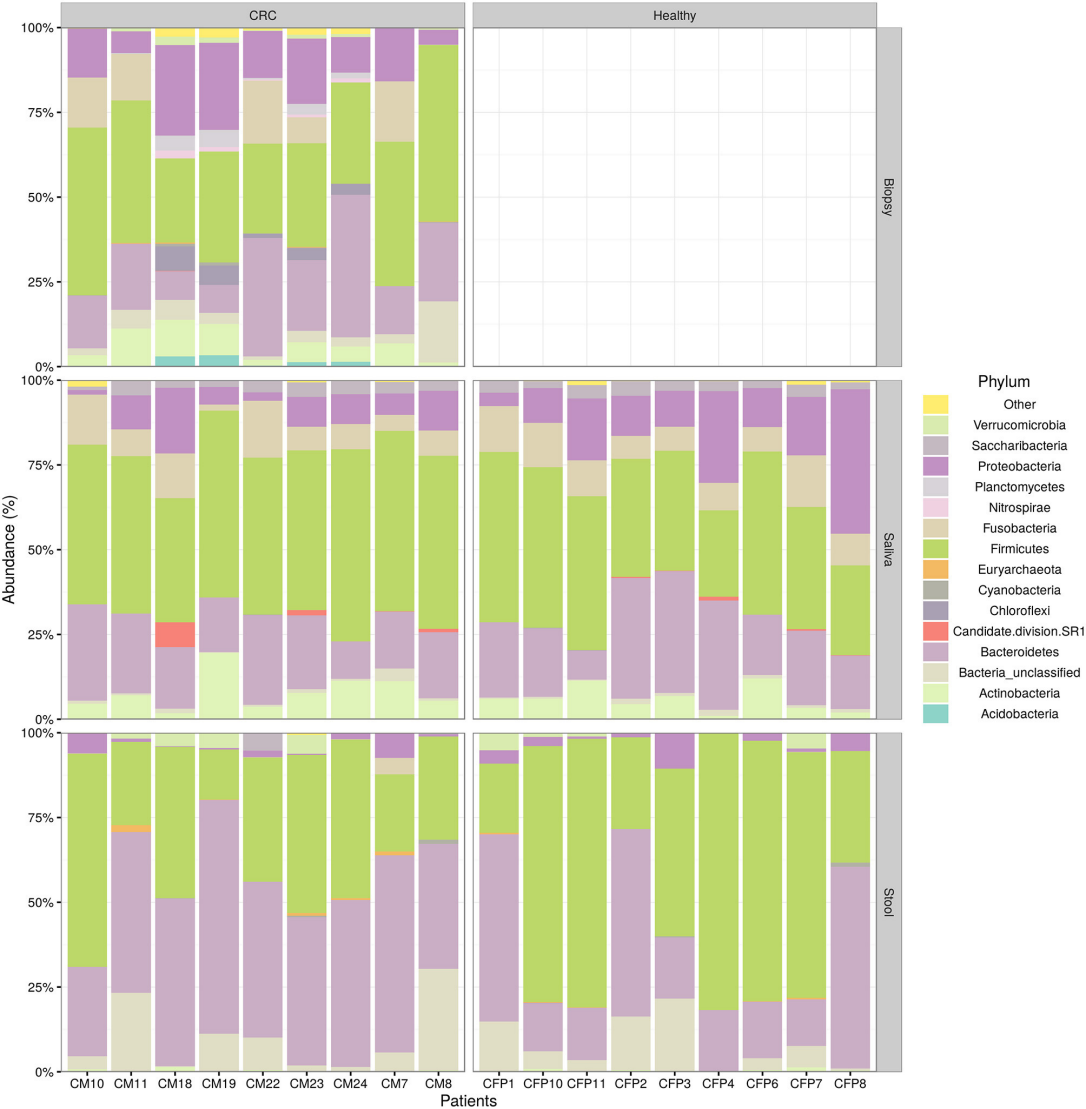
Taxonomic composition of CRC patient (and healthy controls) microbiota. Relative abundances bar plot showing the relative abundance of bacterial phyla in each sample. All phyla representing less than 5% of the total reads analyzed were included in the “Other” group.

**Figure 2:**
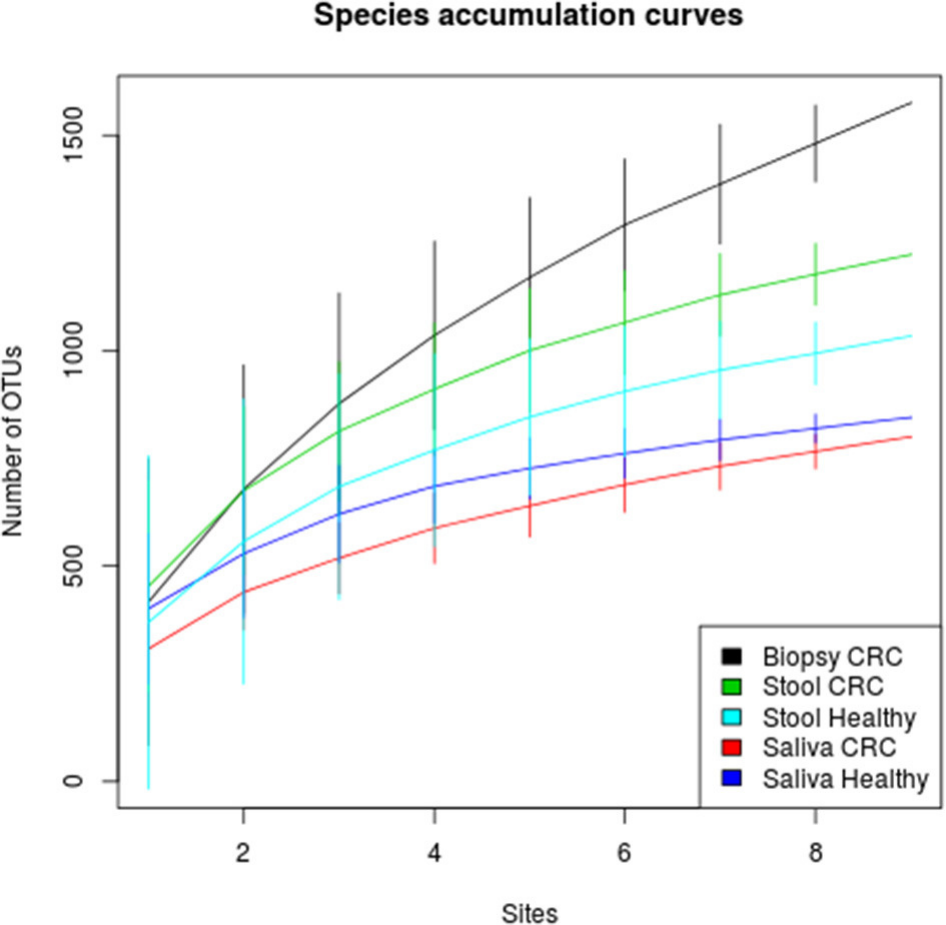
Species accumulation curves of healthy and CRC patients for each sampling body site. CT samples seem to have the highest alpha diversity whereas the bacterial diversity of CRC patients and controls is similar within each body site.

**Figure 3:**
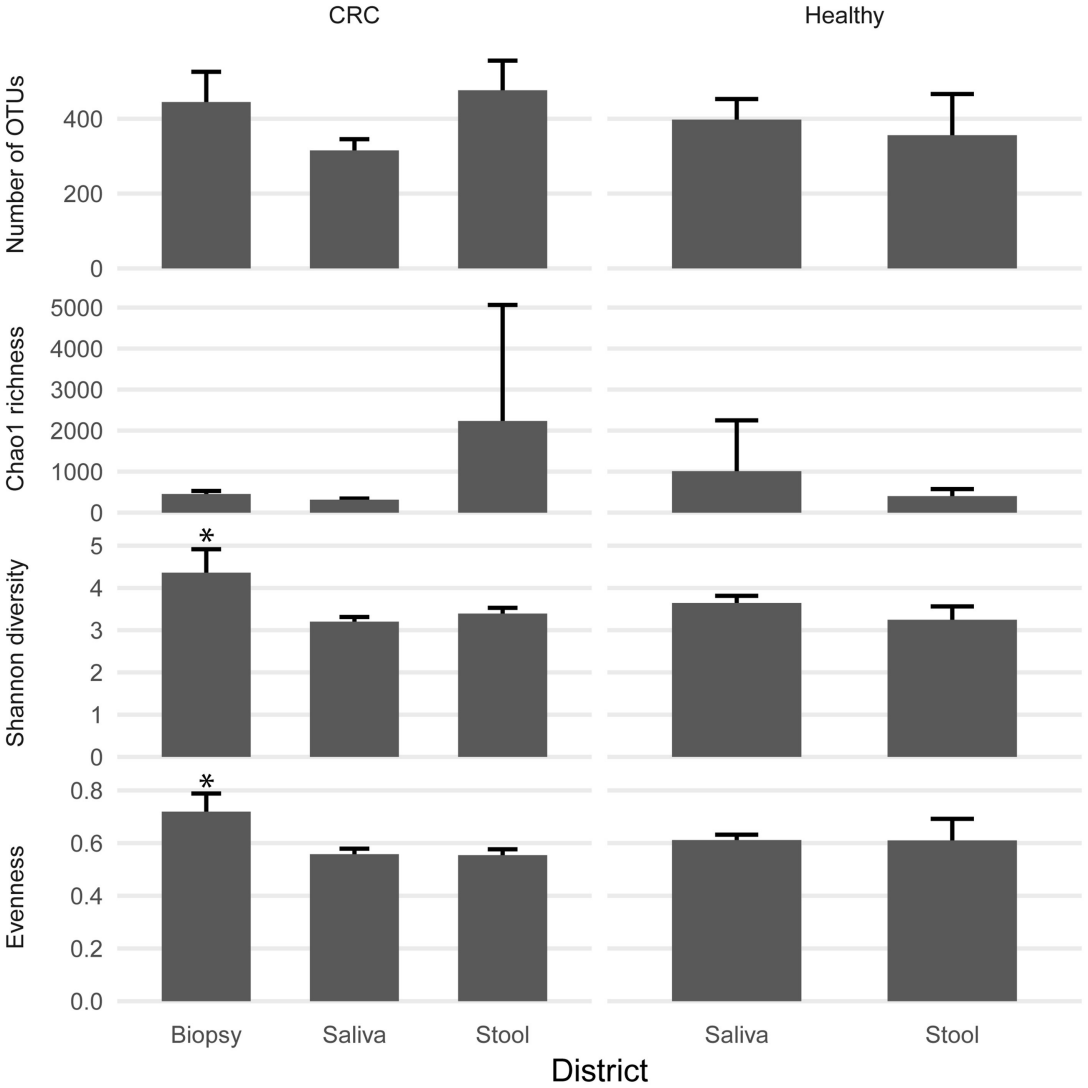
Biodiversity indices distribution according to sampling site and patient status. Patients with CRC and healthy ones reported similar diversity values for all the indices considered. CRC patients showed higher values of alpha diversity in biopsy samples. Asterisks indicate a significant Kruskal-Wallis test with alpha = 0.05.

**Figure 4:**
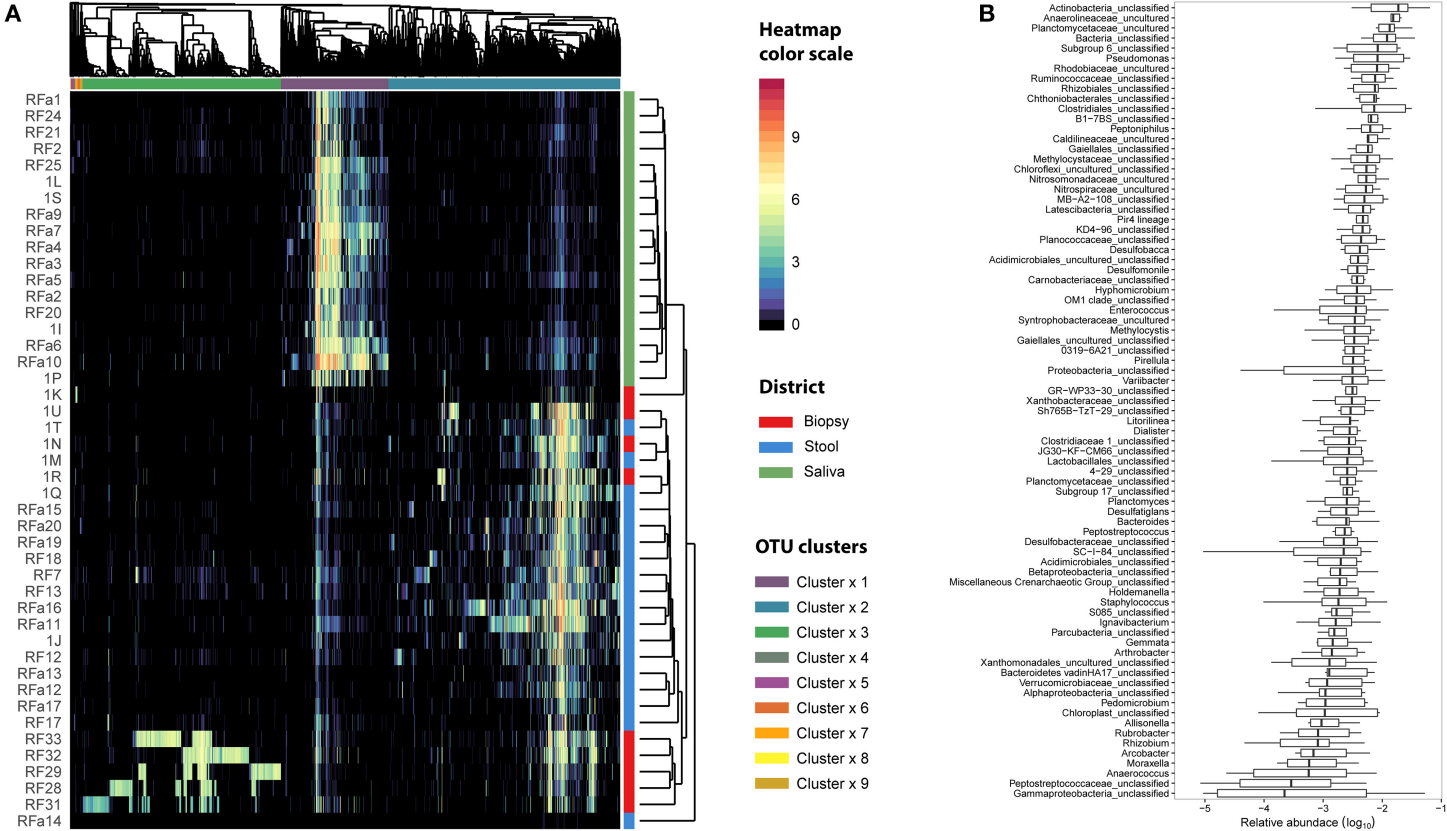
Microbiota distribution for each analyzed sample. **(A)** The number of reads assigned to each OTU was log transformed and reported using the color scale whereas sampling body sites were reported with different colors (red for biopsy samples, blue for stool samples, and green for saliva samples). Dendrograms were produced with average clustering method (UPGMA) based on Bray-Curtis distance. Clusters of OTUs were assessed cutting the dendrogram at 0.95 height. **(B)** The relative abundance of OTUs belonging to cluster 3 was collapsed at Genus level taking into account only the biopsy samples. Genera with an abundance lower than 1% were removed from the analysis. Relative abundance was computed dividing the number of 16S sequences assigned to each OTU by the total number of sequences obtained for each sample. Boxes denote the interquartile range (IQR) between the 25th and the 75th percentile (first and third quartiles), whereas the inner line represents the median. Whiskers represent the lowest and highest values within 1.5 times IQR from the first and third quartiles Outliers were reported using white circles.

We examined the oral cavity and gut (feces and cancer tissue) bacterial communities of 10 patients with CRC and 10 age- and healthy-controls (Table S1). A dataset of 8,595,385 high-quality 16S rRNA sequences (*n* = 20 patient and control fecal samples, *n* = 20 patient and control saliva samples, *n* = 10 CT samples) was obtained. The final data set included 574 bacterial genera belonging to 36 different phyla with a different distribution across samples (Figure 1). Samples reported a Good's coverage estimator ranging from 99 to 100% indicating that roughly 1% of the reads in a given sample came from OTUs that appear only once in that sample (Table S3). The alpha diversity of samples from healthy patients and samples from CRC patients did not report significant differences. However, at body site level differences were observed. In particular, biopsy samples showed a higher level of both Shannon index and Evenness (Kruskal-Wallis test, χ^2^ = 9.9, *p*-values = 0.007 for Shannon index, and χ^2^ = 10.9, *p*-values = 0.004 for Evenness). The highest value of Evenness in biopsy samples together with the highest value of Shannon index revealed a high complexity of this environment. The analysis of the taxonomic composition revealed that more than 85% of the sequences collected were classified into five phyla: *Firmicutes* (39.18%), *Bacteroidetes* (30.36%), *Proteobacteria* (10.65%), *Fusobacteria* (5.15%), and *Actinobacteria* (4.22%). Differences in bacterial community composition were assessed using the analysis of similarity (ANOSIM function with 1,000 permutations). The different body sites showed different bacterial compositions (*R* = 0.834, *P* = 0.001), along with feces samples coming from controls and CRC patients (*R* = 0.201, *P* = 0.005). CT samples exhibited a composition of bacterial communities different from that present in stool samples in CRC patients (*R* = 0.61, *P* = 0.001). Accumulation curves reported in Figure 2 showed a different biodiversity level across body sites. In particular biopsy samples of patients affected by CRC were the ones reporting the highest level of bacterial diversity as confirmed by Kruskall-Wallis test reported in Figure 3. Hierarchical cluster analysis based on the log transformed abundances of OTUs showed that saliva samples are clustered together confirming that there is no statistical difference between the microbiota of CRC and healthy patients inhabiting this compartment (ANOSIM analysis: *R* = 0.049, *P* = 0.176). CT samples showed the highest alpha diversity value compared to other body sites, whereas the taxonomic diversity of samples from CRC and healthy patients was similar within the same body site (Table S4 and Figure 3). In contrast, CT samples seem to have a distinct pattern of bacterial distribution as reported in Figure 4 (cluster 3) with a wide distribution of bacterial genera belonging to the major phyla previously detected (*Proteobacteria, Bacteroidetes*, and *Firmicutes*).

### qPCR assay and association of *F. nucleatum* colonization with clinicopathologic features of CRC patients

**Figure 5:**
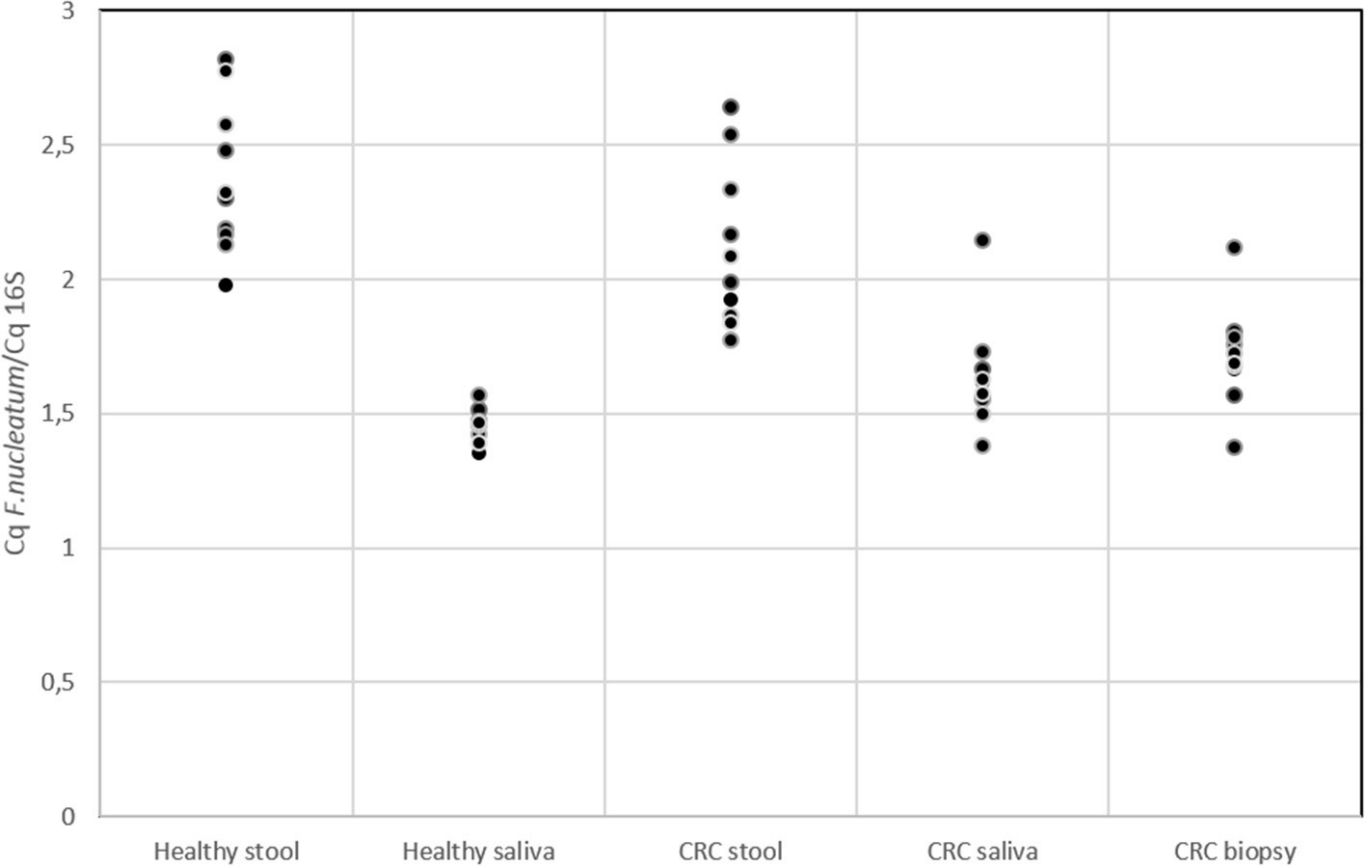
qPCR results for quantification of *F. nucleatum* respect to the total bacterial community; quantification is expressed as the ratio between the Cq value of *F. nucleatum* 16S rRNA gene and the Cq value of the total bacterial community 16S rRNA gene amplicons. The higher is the ratio value, the lowest is the quantification of the target amplicon in the sample.

Several studies in literature suggested the role of *F. nucleatum* as cancer-promoting bacteria (Castellarin et al., 2012; Kostic et al., 2012; Flanagan et al., 2014; Ito et al., 2015; Ramos and Hemann, 2017). Members of *Fusobacterium* genus were detected through 16S rRNA analysis but this approach did not allow us to reach species resolution; thus we decided to further investigate the distribution of this genus using qPCR to confirm the presence of members of *F. nucleatum* species. In Figure 5, the quantification of *F. nucleatum* expressed as the ratio between the Cq value of *F. nucleatum* 16S rRNA gene, and the Cq value of the total bacterial community 16S rRNA gene amplicons is reported. Consequently, the lowest ratio values indicate higher *F. nucleatum* abundance in the samples. No significant differences between stool samples of healthy subjects and stool samples of CRC patients were observed (Table S5). Moreover, neither statistically significant differences were evidenced between saliva samples of healthy and CRC subjects. However, results highlighted that *F. nucleatum* abundance is higher in saliva than stool samples both in healthy subjects (*p* < 0.002) and in CRC patients (*p* < 0.01).

**Figure 6:**
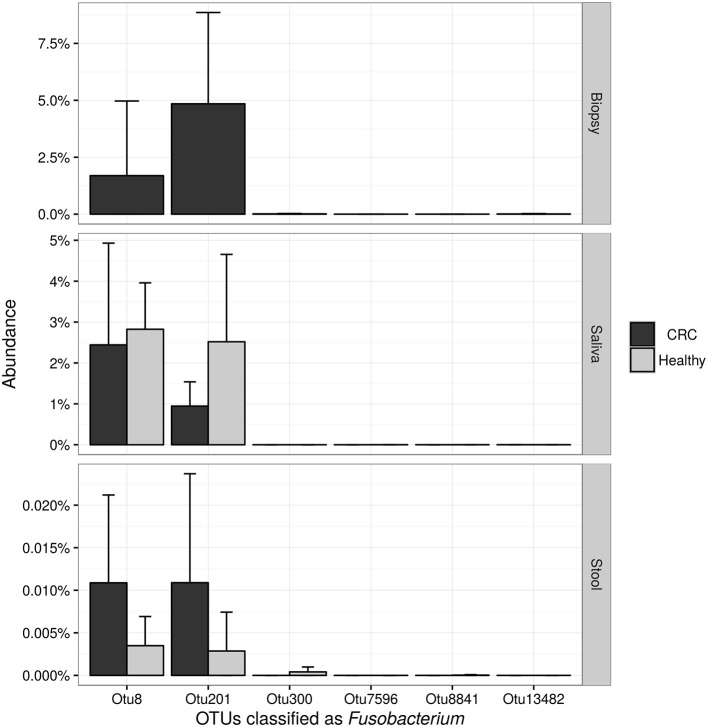
Barplots reporting the average number of sequences assigned to each OTU classified as Fusobacterium. Error bars represent the 95% confidence interval of the distribution.

NGS 16S rRNA data were then analyzed, taking into account all OTUs assigned to *Fusobacterium* genus. A search over the GenBank database by using the BLAST algorithm sorted out that all but OTU 300, matched with *F. nucleatum* sequences in the top-10 hits with an identity threshold ≥95%, confirming the feasibility of using NGS data for *F. nucleatum* DNA detection in our samples. Obtained results (Figure 6) confirmed qPCR data, revealing that stool samples had the lowest number of reads assigned to this genus in contrast with CT samples, which showed the highest number of sequences related to *Fusobacterium* genus. Neither NGS data were underlining differences between healthy controls and CRC patients.

### Distinct taxonomic distribution in different body sites

**Figure 7:**
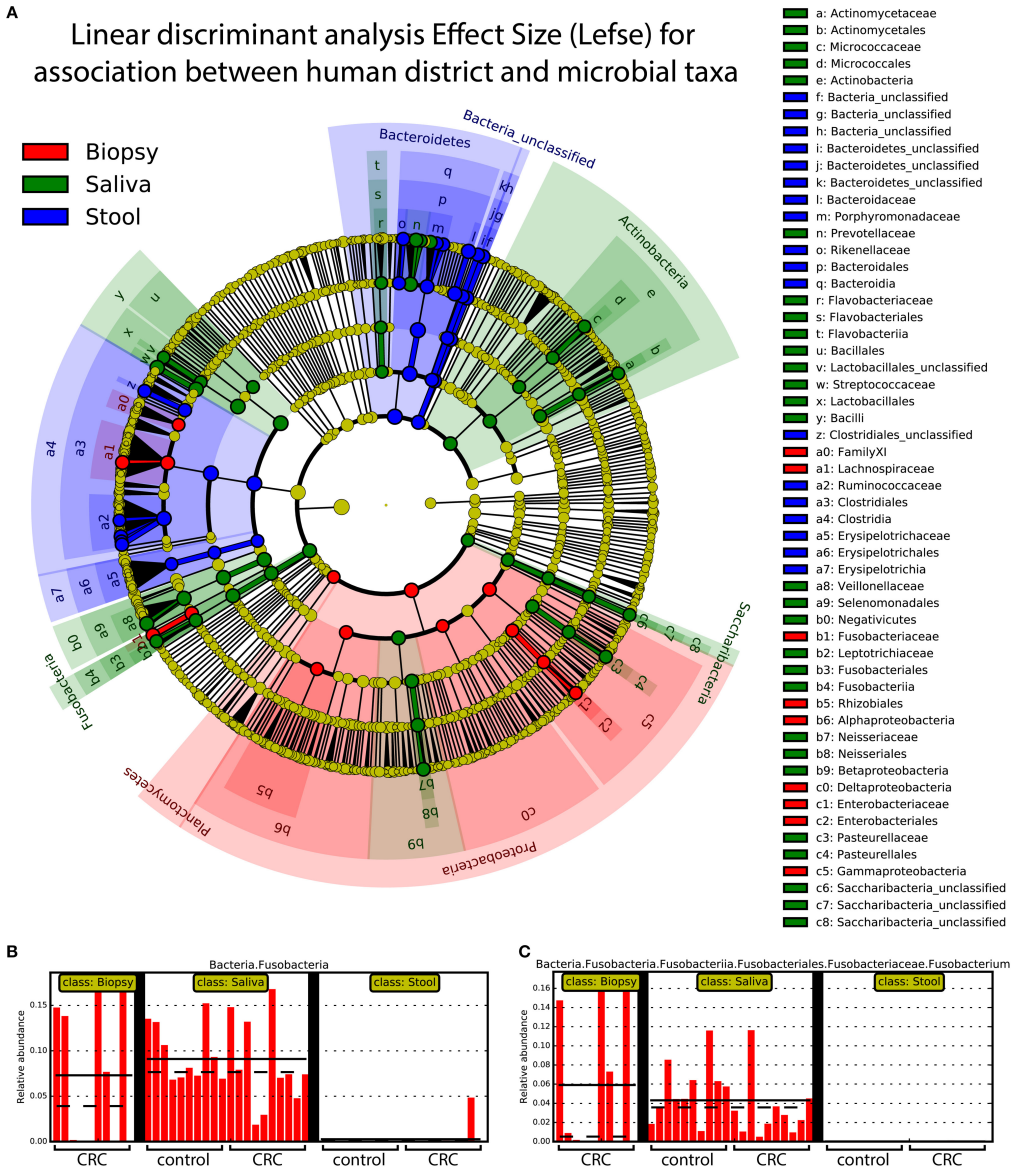
Linear discriminant analysis of association between sampling sites and microbial taxa. A linear discriminant analysis was performed using Lefse and considering the three body sites sampled in this study, namely: biopsy (red), saliva (green), and stool (blue). **(A)** Different body sites showed a characteristic taxonomic composition with major clades strongly associated with a particular site. *Proteobacteria* were mostly associated with biopsy samples whereas *Fusobacteria* and *Bacteroidetes* were mainly associated with saliva and stool samples, respectively. **(B)** Saliva samples reported highest values of *Fusobacteria* members even if **(C)** *Fusobacterium* genus was mainly found in biopsy samples. Each body site has been represented using different colors **(A)** whereas the relative abundance of *Fusobacteria* **(B)** and *Fusobacterium* clade **(C)** was reported for each subject. Samples coming from control and CRC patients were reported below **(B,C)**.

Linear discriminant analysis revealed a distinct microbial distribution in regard of sampling site (Figure 7A). In particular, saliva samples were mainly enriched in members of *Actinobacteria, Saccharibacteria, Proteobacteria* (Beta class), *Fusobacteria, Firmicutes* (mainly *Negativicutes* and *Bacilli*), and *Bacteroidetes* (exclusively represented by members of *Flavobacteriia* class and *Prevotellaceae* family) whereas stool samples presented a characteristic distribution of *Bacteroidetes* (in particular the *Bacteroidia* class) and *Firmicutes* (mainly *Clostridia* and *Erysipelotrichia* classes). Interestingly, biopsy samples were characterized by members of *Proteobacteria*, mainly Delta, Alpha, and Gamma classes, *Planctomycetes*, and *Firmicutes* (namely *Lachnospiraceae* family and *Clostridiales* family XI). Despite the increased abundance of members of Fusobacteria in saliva samples (Figure 7B), biopsy samples were enriched in members of Fusobacterium genus (Figure 7C). Interestingly, only one OTU classified as *Fusobacterium* was detected in cluster 3 (Figure 4A) reporting a mean abundance lower than 1%. On the contrary, collapsed counts based on shared taxonomic classifications showed a higher presence of members of *Fusobacterium* genus (2.9% in the whole dataset) and an even higher abundance in biopsy samples (5.9%, Figure 7C).

## Discussion

### Comparison of oral and intestinal microbiota in CRC patients vs. healthy controls

The aim of this study was to explore, characterize, and compare the bacterial community composition in different body compartments (saliva, stool, and cancer tissue) of CRC Italian patients, through the NGS analysis. Data obtained revealed that the bacterial communities of the three analyzed body sites of both CRC patients and healthy individuals differed significantly in terms of bacterial community composition: the analysis of similarity showed different bacterial taxonomic compositions in saliva, stool, and biopsy. In addition, also linear discriminant analysis (performed with the “lefse“ pipeline to detect distinctive taxa of saliva, feces, and biopsy) revealed a distinct microbial distribution with respect to sampling sites. According to previous studies (Gao et al., 2015), the relative richness values of dominant phyla *Bacteroidetes, Firmicutes*, and *Proteobacteria* were all different in both CRC patients and healthy controls.

Saliva is a biological fluid that could be suitable for biomarker detection. The average adult produces more than 1,000 ml of saliva per day, which always flows into the gastrointestinal tract, thus, the salivary microbiota affects the development of intestinal microbiota in some respects. Our study shows that the microbial community of saliva is, as expected, very different from that inhabiting the other two body sites of the same patient. Variations of the salivary microbiota composition in oral health and disease conditions have been previously described (Belstrøm, 2015), suggesting that the oral microbial compositions may theoretically reflect the oral and general health status. According to previous data (Aas et al., 2005; Keijser et al., 2008; Lazarevic et al., 2009; Nasidze et al., 2009; Bik et al., 2010), we observed that sequences affiliated to the phylum *Firmicutes* dominates the bacterial communities in saliva samples, even though no significant difference was found between CRC patients and healthy controls The application of the linear discriminant analysis, showed an enrichment in members of *Actinobacteria, Saccharibacteria, Proteobacteria* (Beta class), *Fusobacteria, Firmicutes* (mainly *Negativicutes* and *Bacilli*), and *Bacteroidetes* (exclusively represented by members of *Flavobacteriia* class and *Prevotellaceae* family) in saliva samples. In particular, we observed an enrichment of *Fusobacteria*, in agreement with previously reported data (Bolstad et al., 1996). Stool samples exhibited a characteristic distribution of *Bacteroidetes* (in particular the *Bacteroidia* class) and *Firmicutes* (mainly *Clostridia* and *Erysipelotrichia* classes).

Our data suggest that bacterial communities and CRC might be further investigated for their possible correlations in order to assess the opportunity of detecting colon cancer through the analysis of specific fecal bacterial markers. In particular, the fecal-associated microbiota could be dynamically linked to colon cancer, which, in turn, may offer evidence for microflora-associated diagnostic, preventive, prognostic and therapeutic approaches for CRC. However, it is clear that additional studies will be required before a complete examination of these findings.

Because of the prognostic relevance of CRC detection and the prospective testing of fecal samples for bacterial biomarkers, we evaluated whether fecal microbiota profiles mirrored those findings for the colon mucosa tissue. For ethical reasons, healthy intestinal biopsies were not included in the pilot study; accordingly, only few studies evaluating the CRC mucosal microbial community included healthy individuals as controls (Dejea et al., 2014; Geng et al., 2014). As expected, we found that the microbiota compositions in neoplastic tissue significantly diverge from the intestinal lumen (stool) one, in agreement with Flemer et al. (2017). The relative abundance of dominant phyla *Firmicutes, Bacteroidetes, Proteobacteria, Fusobacteria* were all different in the two examined body sites (cancer tissue and stool). In particular, *Proteobacteria* and *Fusobacteria* were more abundant in cancer specimens, while a significantly higher abundance of *Firmicutes* and *Fusobacteria* were observed in the stools of CRC patients.

### *F. nucleatum* colonization in patients with CRC vs. healthy controls

The presence of *F. nucleatum* in saliva, tumor tissue, and stool samples was investigated. It is not yet clear whether the presence of particular bacteria (not yet known) in the neoplastic microenvironment is indicative of a causative role in the CRC genesis and development (Gao et al., 2015). It has been previously suggested that the intestinal dysbiosis resulted in different types of colorectal pathologies, as well as CRC, but no specific direct link with the presence of this bacterium has been established (Tremaroli and Bäckhed, 2012; Allen-Vercoe and Jobin, 2014). Recently, NGS investigations provided much significant evidence in this CRC field, especially about the association of *F. nucleatum* with CRC. This bacterium is not a predominant species in stool samples and has been observed in cancer biopsies of CRC subjects by two independent research groups. Using whole-genome sequencing techniques, an enrichment of *Fusobacterium* spp. was observed, DNA sequences were detected in CT samples compared with the control ones (Kostic et al., 2012). Notably, among them, the most represented phylotype was *F. nucleatum*. On the other hand, using RNA-sequencing approaches, the group of Castellarin et al. (2012) detected a richness of *Fusobacterium* spp. in CRC vs. healthy tissues. On these premises, the two research groups have proposed a direct link between this bacterium and CRC pathogenesis. *F. nucleatum* is commonly found in periodontal plaque and, in the oral cavity, it is associated with viruses, which adhere to host tissue cells influencing the host's immune response (Bolstad et al., 1996). As expected qPCR data (Figure 5) highlighted that in saliva samples the presence of *F. nucleatum* is higher (with respect to total bacterial) than in stool (Bolstad et al., 1996). However, we cannot *a priori* exclude the possibility that our analysis detected also a mixture of closely related (and potentially still unknown) *Fusobacterium* strains/species. *F. nucleatum* is a key player in modifying intestinal inflammation levels (Rubinstein et al., 2013). In addition, in a study about colorectal adenomas, the abundance of *F. nucleatum* was found to positively correlate with inflammatory cytokine gene expression such as TNF (McCoy et al., 2013). TNF-α is produced during the inflammatory response and can promote survival, attachment, and proliferation of metastatic colon cancer cells in a mouse model of lung metastasis depending on the activation of NF-κB by inflammation and cancer cells (Luo et al., 2004). Moreover, through activation of NF-κB and STAT3, TNF-α can enhance epithelial-mesenchymal transition which are critical steps that allow polarized epithelial tumor cells to become mesenchymal like, enhancing cell migration and invasion (Yang and Weinberg, 2008). In light of possible roles of *F. nucleatum* in downregulating T cell-mediated antitumor immune responses (Kostic et al., 2013) and in promoting colorectal tumor progression, future investigations on a larger number of patients may be warranted to explore the impact of *F. nucleatum* on the T cell-based immunotherapy efficacy for CRC.

## Conclusions

Here, we demonstrated a different bacterial taxonomic composition in CRC stool samples vs. healthy controls. We are aware that this study may suffer some limitations, such as the restricted number of enrolled patients, the not complete assessment of the oral health status (that might influence the composition of oral microbiota) and the lack of evaluation of other intestinal bacterial species (e.g., *Escherichia coli* and *Bacteroides fragilis*) (Arthur et al., 2012). In spite of this, our pilot study represents the first simultaneous comparison of the microbial compositions of the tree different body sites (saliva, stool, CT samples). Analyzing a single body niche to categorize CRC individuals does not enable researchers to comprehensively study the spatial variations of the microbiota in CRC. Therefore, the proposed strategy of characterizing the spatial community structures of CRC microbiota (according to Zhang et al., 2017) is crucial to improve our understanding of the mutual interplay between microflora and CRC presence.

However, to further validate these findings and to extend our knowledge about the variation of bacterial composition as a function of CRC (and *vice versa*), it will be helpful to account for a larger number of patients with longitudinal time points. This will promote the development of novel microbial-related diagnostic instruments and therapeutic approaches.

## Availability of data and materials

The partial 16S rRNA gene sequence data generated in this study were submitted to the GenBank Sequence Read Archive accession number PRJNA356414.

## Author contributions

ER, RF, AA, and AM designed the research; AT, MR, EN, FR, FM, MM, and RB collected the samples; ER, CC, and CF performed the experiments; GB analyzed the data; ER, GB, CC, and CF wrote the paper; ER, AA, RF, AM, and PB supervised all the experimental work and revised the manuscript; all authors critically read and approved the manuscript.

### Conflict of interest statement

The authors declare that the research was conducted in the absence of any commercial or financial relationships that could be construed as a potential conflict of interest.

## Abbreviations

NGS: Next-Generation Sequencing
CT: cancer tissue
CRC: colorectal cancer
qPCR: Quantitative polymerase chain reaction
OTUs: operational taxonomic units.
