# Supplementary material for: Preliminary Comparison of Oral and Intestinal Human Microbiota in Patients with Colorectal Cancer: A Pilot Study
Source: Front Microbiol. 2018 Jan 12;8:2699. doi: 10.3389/fmicb.2017.02699 (PMC5770402; doi:10.3389/fmicb.2017.02699)
Supplement: Supplementary file 3 [file DataSheet3.DOCX]

|  | Healthy stool | Healthy saliva | CRC stool | CRC saliva | CRC biopsy |
| --- | --- | --- | --- | --- | --- |
| Healthy stool |  |  |  |  |  |
| Healthy saliva | 0,001827 |  |  |  |  |
| CRC stool | 0,6402 | 0,001827 |  |  |  |
| CRC saliva | 0,003298 | 0,05795 | 0,01008 |  |  |
| CRC biopsy | 0,002461 | 0,02827 | 0,01706 | 1 |  |

Table S3: Mann-Whitney pairwise post-hoc tests; bonferroni corrected *p* values: The values shown are *p*’ = *pNp*. Marked as significant if *p*’<0.05.
