# Supplementary material for: Preliminary Comparison of Oral and Intestinal Human Microbiota in Patients with Colorectal Cancer: A Pilot Study
Source: Front Microbiol. 2018 Jan 12;8:2699. doi: 10.3389/fmicb.2017.02699 (PMC5770402; doi:10.3389/fmicb.2017.02699)
Supplement: Supplementary file 6 [file DataSheet6.DOCX]

| Subject ID | Status | District | Number  of  OTUs | Number  of singletons | Number  of doubletons | Chao1 richness | Shannon diversity | Evenness | Good’s coverage estimator |
| --- | --- | --- | --- | --- | --- | --- | --- | --- | --- |
| CM10 | CRC | Biopsy | 354 | 76 | 26 | 459.56 | 4.21 | 0.72 | 99.93 |
| CM11 | CRC | Biopsy | 386 | 80 | 24 | 512.40 | 3.94 | 0.66 | 99.98 |
| CM18 | CRC | Biopsy | 461 | 48 | 8 | 506.33 | 5.26 | 0.86 | 99.65 |
| CM19 | CRC | Biopsy | 495 | 38 | 12 | 444.08 | 5.35 | 0.86 | 99.71 |
| CM20 | CRC | Biopsy | 495 | 33 | 12 | 411.62 | 5.33 | 0.86 | 99.78 |
| CM22 | CRC | Biopsy | 441 | 30 | 15 | 425.19 | 3.16 | 0.52 | 99.93 |
| CM23 | CRC | Biopsy | 612 | 46 | 16 | 555.88 | 5.22 | 0.81 | 99.73 |
| CM24 | CRC | Biopsy | 658 | 66 | 14 | 661.00 | 4.36 | 0.67 | 99.83 |
| CM7 | CRC | Biopsy | 132 | 0 | 0 | 132.00 | 2.73 | 0.56 | 100.00 |
| CM8 | CRC | Biopsy | 413 | 49 | 38 | 443.15 | 4.04 | 0.67 | 99.96 |
| CM10 | CRC | Saliva | 275 | 0 | 0 | 275.00 | 3.29 | 0.58 | 100.00 |
| CM11 | CRC | Saliva | 283 | 0 | 0 | 283.00 | 3.40 | 0.60 | 100.00 |
| CM18 | CRC | Saliva | 300 | 0 | 0 | 300.00 | 3.07 | 0.54 | 100.00 |
| CM19 | CRC | Saliva | 300 | 0 | 0 | 300.00 | 3.00 | 0.53 | 100.00 |
| CM20 | CRC | Saliva | 382 | 0 | 0 | 382.00 | 3.11 | 0.52 | 100.00 |
| CM22 | CRC | Saliva | 236 | 0 | 0 | 236.00 | 3.01 | 0.55 | 100.00 |
| CM23 | CRC | Saliva | 360 | 0 | 0 | 360.00 | 3.53 | 0.60 | 100.00 |
| CM24 | CRC | Saliva | 332 | 0 | 0 | 332.00 | 2.91 | 0.50 | 100.00 |
| CM7 | CRC | Saliva | 385 | 0 | 81 | 385.00 | 3.37 | 0.57 | 100.00 |
| CM8 | CRC | Saliva | 301 | 0 | 55 | 301.00 | 3.33 | 0.58 | 100.00 |
| CFP1 | Healthy | Saliva | 295 | 0 | 0 | 295.00 | 3.49 | 0.61 | 100.00 |
| CFP10 | Healthy | Saliva | 413 | 0 | 101 | 413.00 | 3.77 | 0.63 | 100.00 |
| CFP11 | Healthy | Saliva | 516 | 77 | 47 | 576.96 | 3.97 | 0.64 | 99.99 |
| CFP2 | Healthy | Saliva | 243 | 0 | 0 | 243.00 | 3.58 | 0.65 | 100.00 |
| CFP3 | Healthy | Saliva | 351 | 0 | 93 | 351.00 | 3.60 | 0.61 | 100.00 |
| CFP4 | Healthy | Saliva | 371 | 0 | 71 | 371.00 | 3.58 | 0.61 | 100.00 |
| CFP6 | Healthy | Saliva | 462 | 0 | 137 | 462.00 | 3.58 | 0.58 | 100.00 |
| CFP7 | Healthy | Saliva | 537 | 110 | 0 | 6532.00 | 4.17 | 0.66 | 99.94 |
| CFP8 | Healthy | Saliva | 409 | 67 | 32 | 476.00 | 3.15 | 0.52 | 99.98 |
| CFP9 | Healthy | Saliva | 380 | 0 | 101 | 380.00 | 3.54 | 0.60 | 100.00 |
| CM10 | CRC | Stool | 448 | 63 | 31 | 509.03 | 3.84 | 0.63 | 99.95 |
| CM11 | CRC | Stool | 423 | 86 | 0 | 4078.00 | 3.44 | 0.57 | 99.98 |
| CM18 | CRC | Stool | 403 | 0 | 0 | 403.00 | 3.27 | 0.55 | 100.00 |
| CM19 | CRC | Stool | 603 | 0 | 190 | 603.00 | 3.21 | 0.50 | 100.00 |
| CM20 | CRC | Stool | 701 | 167 | 0 | 14562.00 | 3.43 | 0.52 | 99.97 |
| CM22 | CRC | Stool | 247 | 0 | 0 | 247.00 | 3.06 | 0.56 | 100.00 |
| CM23 | CRC | Stool | 483 | 0 | 123 | 483.00 | 3.40 | 0.55 | 100.00 |
| CM24 | CRC | Stool | 656 | 0 | 178 | 656.00 | 3.61 | 0.56 | 100.00 |
| CM7 | CRC | Stool | 407 | 0 | 91 | 407.00 | 3.60 | 0.60 | 100.00 |
| CM8 | CRC | Stool | 389 | 0 | 72 | 389.00 | 3.06 | 0.51 | 100.00 |
| CFP1 | Healthy | Stool | 614 | 110 | 36 | 776.03 | 3.65 | 0.57 | 99.97 |
| CFP10 | Healthy | Stool | 433 | 0 | 0 | 433.00 | 3.00 | 0.49 | 100.00 |
| CFP11 | Healthy | Stool | 392 | 0 | 89 | 392.00 | 3.45 | 0.58 | 100.00 |
| CFP2 | Healthy | Stool | 296 | 0 | 0 | 296.00 | 3.52 | 0.62 | 100.00 |
| CFP3 | Healthy | Stool | 223 | 0 | 0 | 223.00 | 2.84 | 0.52 | 100.00 |
| CFP4 | Healthy | Stool | 8 | 0 | 0 | 8.00 | 1.97 | 0.95 | 100.00 |
| CFP6 | Healthy | Stool | 361 | 0 | 73 | 361.00 | 3.16 | 0.54 | 100.00 |
| CFP7 | Healthy | Stool | 619 | 143 | 32 | 926.67 | 4.04 | 0.63 | 99.95 |
| CFP8 | Healthy | Stool | 208 | 0 | 0 | 208.00 | 3.05 | 0.57 | 100.00 |
| CFP9 | Healthy | Stool | 407 | 0 | 0 | 407.00 | 3.79 | 0.63 | 100.00 |

**S6: Biodiversity indices for each sample.** The principal biodiversity indices were reported for each subject included in the study and for each district sampled. The “Number of clones” corresponds to the number of reads correctly assigned to a given sample. The “Number of OTUs” correspond to the richness, whereas the number of singletons and doubletons is the number of sequences found only once or twice, respectively.
