# Supplementary material for: Preliminary Comparison of Oral and Intestinal Human Microbiota in Patients with Colorectal Cancer: A Pilot Study
Source: Front Microbiol. 2018 Jan 12;8:2699. doi: 10.3389/fmicb.2017.02699 (PMC5770402; doi:10.3389/fmicb.2017.02699)
Supplement: Supplementary file 7 [file DataSheet7.DOCX]

|  | **numDF** | **denDF** | **F-value** | **p-value** |
| --- | --- | --- | --- | --- |
| **Number of OTUs** |  |  |  |  |
| (Intercept) | 1 | 28 | 322.587 | 0.0000 |
| district | 2 | 28 | 1.878 | 0.1716 |
| status | 1 | 18 | 0.157 | 0.6963 |
|  |  |  |  |  |
| **Chao1 richness** |  |  |  |  |
| (Intercept) | 1 | 28 | 7.759 | 0.0095 |
| district | 2 | 28 | 0.655 | 0.5270 |
| status | 1 | 18 | 0.642 | 0.4336 |
|  |  |  |  |  |
| **Shannon diversity** |  |  |  |  |
| (Intercept) | 1 | 28 | 2171.472 | 0.0000 |
| district | 2 | 28 | 13.529 | 0.0001 |
| status | 1 | 18 | 0.751 | 0.3976 |
|  |  |  |  |  |
| **Evenness** |  |  |  |  |
| (Intercept) | 1 | 28 | 2629.022 | 0.0000 |
| district | 2 | 28 | 10.432 | 0.0004 |
| status | 1 | 18 | 4.228 | 0.0546 |

**S7: Results of mixed-effect models on biodiversity indices.** The table reports the results of the random intercept models for each biodiversity index. The name of the index is reported in bold whereas the factor name is reported using the “district” and “status” labels referring to the different sampling sites and the patient condition, respectively. numDF, numerator degrees of freedom; denDF, denominator degrees of freedom; F-value, result of the F-test; p-value, significance level of the test.
