# Supplementary material for: Preliminary Comparison of Oral and Intestinal Human Microbiota in Patients with Colorectal Cancer: A Pilot Study
Source: Front Microbiol. 2018 Jan 12;8:2699. doi: 10.3389/fmicb.2017.02699 (PMC5770402; doi:10.3389/fmicb.2017.02699)
Supplement: Supplementary file 8 [file DataSheet8.DOCX]

Supplementary Tables

**Table S1:** Clinical parameter of patients, data collected included anthropometric measurements (height, weight), nutritional data (including the use of probiotics), clinical history and status and medication history.

**Table S2:** Number of sequences obtained after each step of analysis. Percentage values have been computed based on the initial number of pairs.

**Table S3**: Mann-Whitney pairwise post-hoc tests; bonferroni corrected p values: The values shown are p’ = pNp. Marked as significant if p’<0.05.

**Table S4:** Multiple linear regression to predict the abundance of OTUs assigned to *Fusobacterium* genus based on patient's sex, age, height, and body mass index. A different model has been calculated for each OTU reporting coefficients (Coef), standard errors (S.E.), t values (t), and p values (p). P values lower than 0.05 where highlighted with an asterisk.

**Table S5:** One-way analysis of variance ANOVA to compare the effect of T (describes the size of the original (primary) tumor and whether it has invaded nearby tissue) and N (describes regional lymph nodes that are involved) on the abundance of OTUs assigned to *Fusobacterium* genus. A model was computed for each OTU reporting the sum of squares (Sum. Sq.), mean squares (mean sq.), F values (F), and p values (p). P values lower than 0.05 where highlighted with an asterisk.

**Table S6:** Biodiversity indices for each sample. The principal biodiversity indices were reported for each subject included in the study and for each district sampled. The “Number of clones” corresponds to the number of reads correctly assigned to a given sample. The “Number of OTUs” correspond to the richness, whereas the number of singletons and doubletons is the number of sequences found only once or twice, respectively.

**Table S7:** Results of mixed-effect models on biodiversity indices. The table reports the results of the random intercept models for each biodiversity index. The name of the index is reported in bold whereas the factor name is reported using the “district” and “status” labels referring to the different sampling sites and the patient condition, respectively. numDF, numerator degrees of freedom; denDF, denominator degrees of freedom; F-value, result of the F-test; p-value, significance level of the test.
